# Developing a nomogram based on SEER database for predicting prognosis in choroid plexus tumors

**DOI:** 10.1038/s41598-024-63218-1

**Published:** 2024-05-28

**Authors:** Zedi Yang, Heng Jiang, Ding He, Sheng Zhang, Lei Huang, Peigeng Gao, Haiyan Huang, Junguo Cao, Zhixin Zhan

**Affiliations:** 1 Department of Neurosurgery, The First Hospital of Jilin University, Changchun, 130021 China; 2 Department of Neurosurgery, The Second Hospital of Jilin University, Changchun, 130041 China; 3 Department of Hepatobiliary and Pancreatic Surgery, General Surgery Center, The First Hospital of Jilin University, Changchun, 130021 China; 4 Department of Neurosurgery, First Affiliated Hospital, School of Medicine, Zhejiang University, Hangzhou, 310003 China

**Keywords:** Choroid plexus tumor, Nomogram, SEER program, Epidemiology, Prognosis, Cancer epidemiology, CNS cancer, Oncology, Risk factors

## Abstract

Choroid plexus tumors (CPT) are rare and highly vascularized neoplasms that have three histologically confirmed diagnoses, including choroid plexus papilloma, atypical choroid plexus papilloma, and choroid plexus carcinoma (CPC). This study aimed to determine the epidemiology and survival of patients with CPTs and develop a nomogram to quantify the prognosis of the patients with CPT. Data of 808 patients who were diagnosed as CPT between 2000 and 2020 was obtained from the surveillance, epidemiology, and end results database. Descriptive analysis was used to assess the distribution and tumor-related characteristics of the patients with CPT. Independent prognostic factors for patients with CPT were identified by univariate and multivariate Cox regression analysis. The nomogram was established and evaluated by receiver operating characteristic curve, and decision curve analysis (DCA), calibration curves. The independent prognostic factors for patients with CPT are age, tumor size, surgery, chemotherapy, tumor number, pathologies, and race. For the prognostic nomogram, the area under the curve (AUC) of 60-, 120-, and 180-months were 0.855, 0.869 and 0.857 in the training set and 0.836, 0.864 and 0.922 in the test set. The DCA and calibration curve indicated the good performance of the nomogram. Patients with CPTs can be diagnosed at any age. Among the three histopathological tumors, patients with CPC had the worst prognosis. The nomogram was established to predict the prognosis of patients with CPT, which had satisfactory accuracy, and clinical utility may benefit for clinical decision-making.

## Introduction

Choroid plexus tumors (CPTs) are rare and highly vascularized neoplasms that account for only 1% of all brain tumors^1^. CPTs originate in the differentiated epithelial tissue of the choroid plexus and occur in the ventricular system; patients may present with hydrocephalus and related clinical signs due to the tumor’s ability to affect the normal circulation of the cerebrospinal fluid^2,3^. According to the latest World Health Organization (WHO) classification, CPTs comprise choroid plexus papillomas (CPPs), atypical choroid plexus papillomas (ACPPs), and choroid plexus carcinomas (CPCs)^1,4^. The age-adjusted incidence rates of CPPs, ACPPs, and CPCs were 0.034, 0.005, and 0.010 per 100,000 person-years, respectively. CPPs are normally benign tumors with a 5-year overall survival (OS) rate of 0.920. By contrast, CPCs are more malignant and invasive^5^. The 5-year overall rate of patients with CPCs was 0.613.

Currently, surgical resection, radiotherapy and chemotherapy are the main treatments for CPT patients. Among them, gross total resection (GTR) is recognized as the most effective therapy for patients with CPTs^1,4,6–10^. Due to the low prevalence of this disease, limited clinical data are available to describe the treatment and prognosis of CPT. In previous studies, many variables affecting prognosis have been identified, including sex, age, ethnicity, pathologic staging, tumor size, surgical extent, radiotherapy, and chemotherapy^2,6–8,11,12^. However, no study has established a predictive model for CPT prognosis, which means that the probability of prognosis cannot be quantified. We need a reliable prognostic model to help physicians make more accurate treatment decisions. Nomogram is a commonly used multivariate visualization tool in oncology that quantitatively predicts each patient’s survival time and risk of recurrence, etc. It can be used to assist in clinical decision-making, helping clinicians develop more effective treatment plans and improve patient survival and quality of life. The National Cancer Institute’s Surveillance, Epidemiology and End Results (SEER) program included 48% of the United States (US) population. Therefore, the present study used publicly available data of the SEER database to analyze the demographics, tumor histology, and treatment of 838 patients with CPTs in the US and created a choroid plexus tumor prognostic nomogram.

## Methods

### Study population selection

The data included in the present study were downloaded from the SEER*Stat software version 8.4.2. The data in the SEER database are available to the public for research purposes. Therefore, our institutional review board grants research exemptions to studies of such data. Patients of any age with histopathological diagnosed CPT were included in the SEER database from 2000 to 2020. The inclusion criteria were following: (1) patients were histologically diagnosed as CPT from 2000 to 2020; (2) demographic variables, including age, race, sex, and marital status were available; (3) tumor characteristics, including histological type, tumor size, and tumor number were available; (4) treatment: surgical type, radiotherapy, and chemotherapy were available. In addition, patients with duplicate patient IDs were excluded from this study. 838 patients were used to form a cohort to study the epidemiological studies of patients with CPT. A new cohort of 808 patients with survival ≥ 1 month was selected. First, we randomized these patients into a training set of 566 (70%) and a test set of 242 (30%). Subsequently, we used the “DMwR” package in the R software to fill in some of the missing fields in the training set and test set respectively, such as surgery (0.88%, 2.07%), race (2.65%, 1.24%), tumor size (22.44%, 22.73%) and marital status (2.30%, 3.31%). In this study, patients in the training set were used to perform prognostic factor studies and develop the nomogram, and patients in the test set were used to validate the nomogram.

### Data collection

In this study, seven variables of age, sex (male, female), race (white, black, other), marital status (married, other), pathologic type (CPP, ACPP, CPC), tumors number (single, multiple), and tumors size (< = 30 mm, > 30 mm), as well as three therapeutic variables of surgery (gross total resection, subtotal resection, no surgery), chemotherapy (yes, no), and radiation therapy (yes, no) were used to study the prognostic factors of patients with CPT.

### Statistical analysis

All statistical analyses in our study were performed using SPSS 26.0 and R software (version 3.6.1). The chi-square test was used to compare variables between the training and test sets. In the present study, a *p* value < 0.05 (two-sided) was considered statistically significant. Kaplan–Meier survival analysis as well as univariate Cox regression analysis were applied to identify prognostic variables. Variables with *p* value < 0.05 in the univariate Cox regression analysis were incorporated in the multivariate Cox regression analysis, and the independent prognostic factors of patients with CPT were identified.

The prognostic nomogram was developed by the “rms” package in R software based on the independent prognostic factors and important clinical factors. The cut-off value of risk score was derived based on the “maxstat” method and patients were categorized into high-risk and low-risk groups. The survival curve with a log-rank test was used to verify the prognostic value of nomogram. Meanwhile, the time-dependent receiver operating characteristic curve for the prognostic nomogram was generated. The area under the curve was used to evaluate the discrimination of this nomogram. In addition, time-dependent ROC curve of all independent variables was also generated, AUCs of all independent variables were compared with the AUC of the nomogram. Moreover, the calibration curves and decision curve analysis curves were established for the nomogram.

### Ethical approval

Cases were collected from the SEER database and were analyzed anonymously; therefore, no additional informed consent was required.

## Results

### Characteristics of the study population

In total, 838 patients with CPTs of any age were identified from 2000 to 2020. CPPs and ACPPs were identified from 2004 to 2020. For CPTs, the age-adjusted incidence rate was 0.04957 cases per 100,000 person-years during the study period. The median age at CPT diagnosis was 19 years. Most patients were diagnosed with CPTs between 2 and 45 years of age. The distribution of the three histological types was as follows: CPP, 69.81%; ACPP, 10.98%; and CPC, 19.21%. The percentage of diagnosed pediatric patients (≤ 2 years) was 28.52% and that of teenage patients (≤ 18 years) was 49.76%. The median ages were 28 years (10–90th percentile 1–66) in CPP cases, 5 years (10–90th percentile 0–61) in ACPP cases, and 2 years (10–90th percentile 0–49) in CPC cases. A total of 421 patients were female (50.2%), and 417 patients were male (49.8%). White people accounted for 82.2% of the total population. In this group, married patients accounted for 23.3% of all patients. Patients who did not undergo surgery accounted for 15.0% of the population, those who received subtotal resection (STR) accounted for 27.9%, patients who received GTR accounted for 55.5%. Patients with tumors ≤ 3 cm accounted for 40.1% of the population, and those with tumors > 3 cm accounted for 36.9%. 55 patients received radiation therapy (6.6%), and 101 patients received chemotherapy (12.1%). Until data collection, there were 708 patients alive (84.5%), and 130 patients died (15.5%). Table 1 shows the demographic and clinical characteristics of the CPP, ACPP, CPC, and CPT groups.

**Table 1:** Comparison of demographic and clinical characteristics of patients diagnosed as CPT.

Characteristic	CPP		ACPP		CPC		CPTs	
	n	%	n	%	n	%	n	%
In total	585	69.8	92	11.0	161	19.2	838	100
Age group (years)								
0	52	8.9	25	27.2	45	28.0	122	14.6
1–4	64	10.9	20	21.7	60	37.3	144	17.2
5–19	130	22.2	9	9.8	19	11.8	158	18.9
20–39	141	24.1	11	12.0	14	8.7	166	19.8
40–59	111	19.0	14	15.2	17	10.6	142	16.9
60–79	72	12.3	13	14.1	5	3.1	90	10.7
80+	15	2.6	0	0.0	1	0.6	16	1.9
Sex								
Female	312	53.3	47	51.1	62	38.5	421	50.2
Male	273	46.7	45	48.9	99	61.5	417	49.8
Race								
White	482	82.4	82	89.1	125	77.6	689	82.2
Black	44	7.5	6	6.5	15	9.3	65	7.8
Other	41	7.0	3	3.3	16	9.9	60	7.2
Unknown	18	3.1	1	1.1	5	3.1	24	2.9
Surgery type								
GTR	303	51.8	55	59.8	107	66.5	465	55.5
STR	169	28.9	27	29.3	38	23.6	234	27.9
No surgery	105	17.9	10	10.9	11	6.8	126	15.0
Unknown	8	1.4	0	0.0	5	3.1	13	1.6
Radiation								
No/unknown	568	97.1	89	96.7	126	78.3	783	93.4
Yes	17	2.9	3	3.3	35	21.7	55	6.6
Chemotherapy								
No/unknown	581	99.3	88	95.7	68	42.2	737	87.9
Yes	4	0.7	4	4.3	93	57.8	101	12.1
Marital status								
Others	427	73.0	74	80.4	142	88.2	643	76.7
Married	158	27.0	18	19.6	19	11.8	195	23.3
Vital status								
Alive	533	91.1	79	85.9	96	59.6	708	84.5
Dead	52	8.9	13	14.1	65	40.4	130	15.5
Tumor size								
≤ 30 mm	280	47.9	29	31.5	27	16.8	336	40.1
> 30 mm	166	28.4	46	50.0	97	60.2	309	36.9
Unknown	139	23.8	17	18.5	37	23.0	193	23.0
Tumor number								
Single	530	90.6	87	94.6	146	90.7	763	91.1
Multiple	55	9.4	5	5.4	15	9.3	75	8.9

### Prognostic factors for patients with CPT

**Figure 1:**
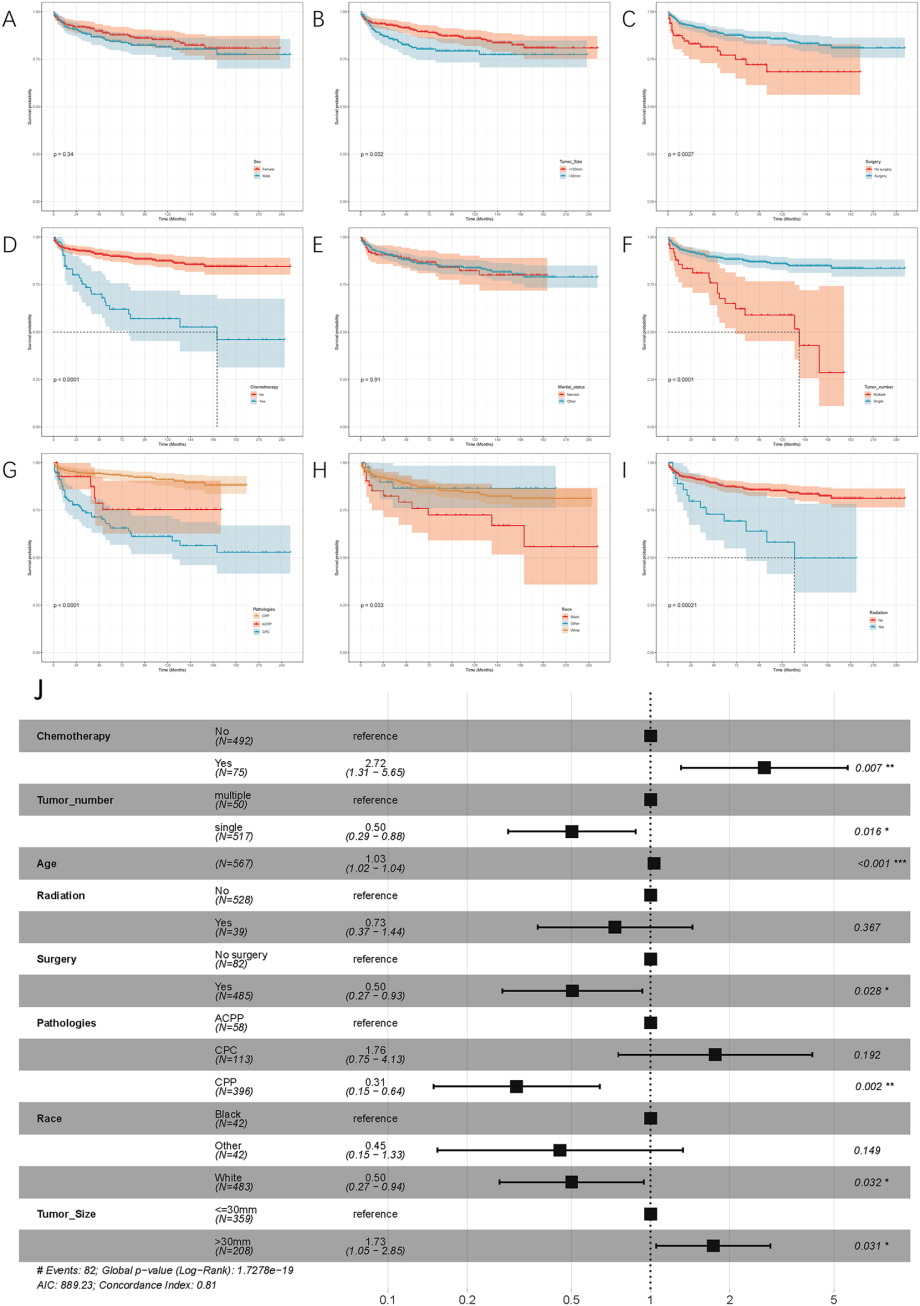
The Kaplan–Meier survival analysis for patients with CPT classified based on (**A**) sex, (**B**) tumour size, (**C**) surgery, (**D**) chemotherapy, (**E**) marital status, (**F**) tumour number, (**G**) pathologies, (**H**) race, and (**I**) radiation. (**J**) Forest plot of multivariate Cox analysis for patients with CPT.

We used Kaplan–Meier and log-rank tests in patients with CPTs to reveal the associations of sex, tumor size, surgery, chemotherapy, marital status, tumor number, pathologies, race, and radiation with OS (Fig. 1A–I). Using the Kaplan–Meier method, we found a significant difference in OS between different histology of CPTs (*p* < 0.001). The 5-year OS rates were 92.0% for CPP, 80.7% for ACPP, and 61.3% for CPC. Table 2 shows the results of univariate Cox regression analysis for the CPT, where age, tumor size, surgery, chemotherapy, tumor number, pathologies, race, and radiation were statistically significant for survival. Combining the results of univariate and multivariate survival analyses (Fig. 1J), age, tumor size, surgery, chemotherapy, tumor number, pathologies, and race had a significant effect on the prognosis of CPT.

**Table 2:** Univariate survival analysis of CPT.

Variable	*p* value	HR	95%CI
Age	< 0.01	1.02	1.01–1.02
Sex			
Female	0.34	0.64	0.42–1.00
Male	0.34	1.60	1.00–2.40
Pathologies			
CPP	< 0.01	0.21	0.13–0.32
ACPP	0.37	1.40	0.70–2.60
CPC	< 0.01	5.2	3.30–8.00
Race			
White	0.01	0.45	0.28–0.74
Black	< 0.01	2.90	1.70–5.00
Other	0.77	1.1	0.49–2.60
Surgery type			
No surgery	< 0.01	2.20	1.30–3.70
Surgery	< 0.01	0.45	0.27–0.75
Radiation			
No	< 0.01	0.25	0.14–0.44
Yes	< 0.01	4.00	2.30–7.00
Chemotherapy			
No	< 0.01	0.21	0.13–0.32
Yes	< 0.01	4.80	3.10–7.60
Marital status			
Others	0.91	0.95	0.57–1.60
Married	0.91	1.00	0.63–1.80
Tumor size			
≤ 3 cm	0.03	0.67	0.43–1.0
> 3 cm	0.03	1.50	0.97–2.30
Tumor number			
Multiple	< 0.01	3.50	2.10–5.70
Single	< 0.01	0.29	0.17–0.47

### Prognostic nomogram for patients with CPT

**Figure 2:**
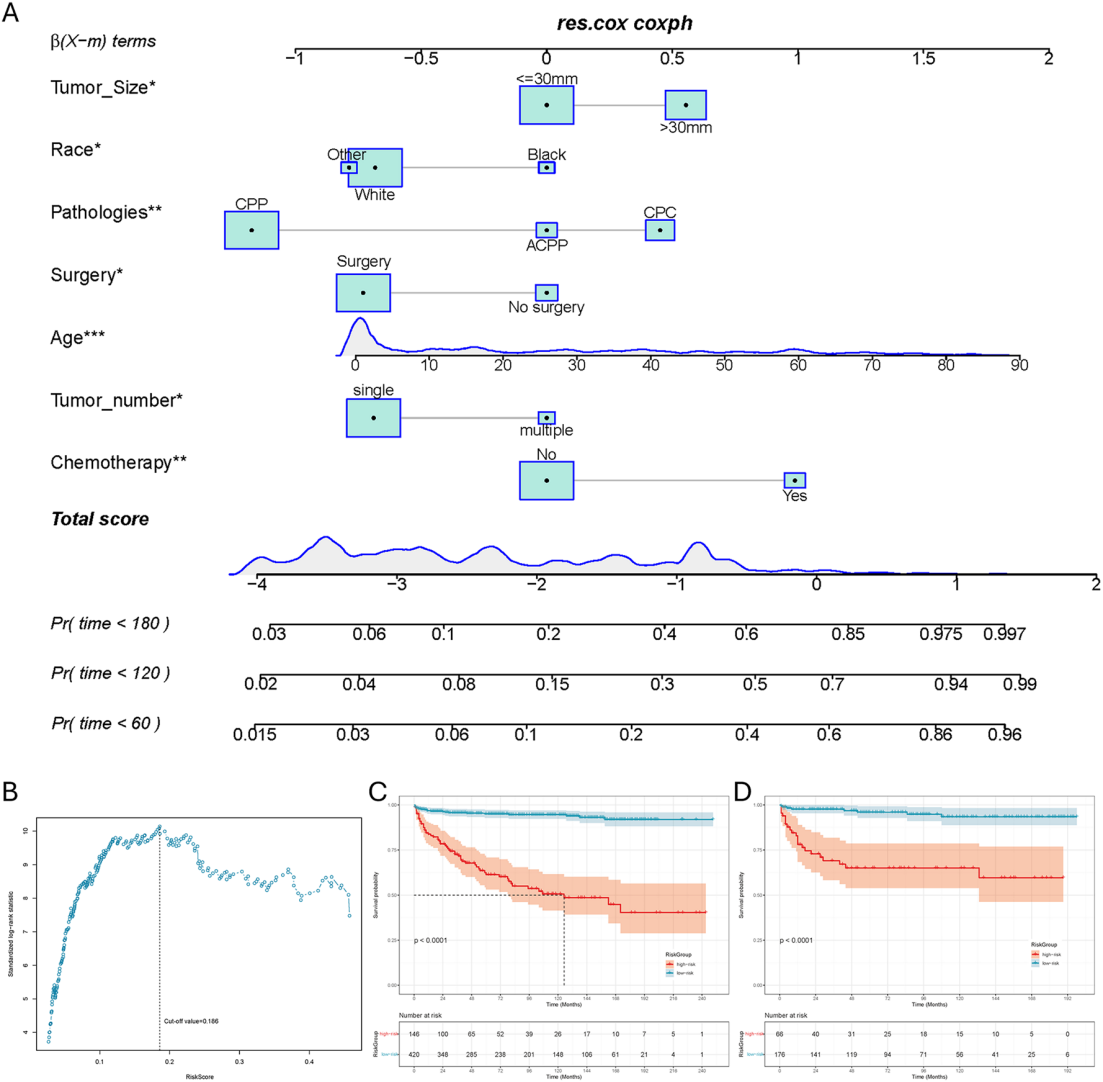
(**A**) A prognostic nomogram for patients with CPT. The area of the box and the area under the curve represent the distribution of the categorical and numerical variables. (**B**) Based on the cutoff value of 0.186, we categorized all patients into low-risk and high-risk groups. The Kaplan–Meier survival curve of the training (**C**) and the testing (**D**) set.

**Figure 3:**
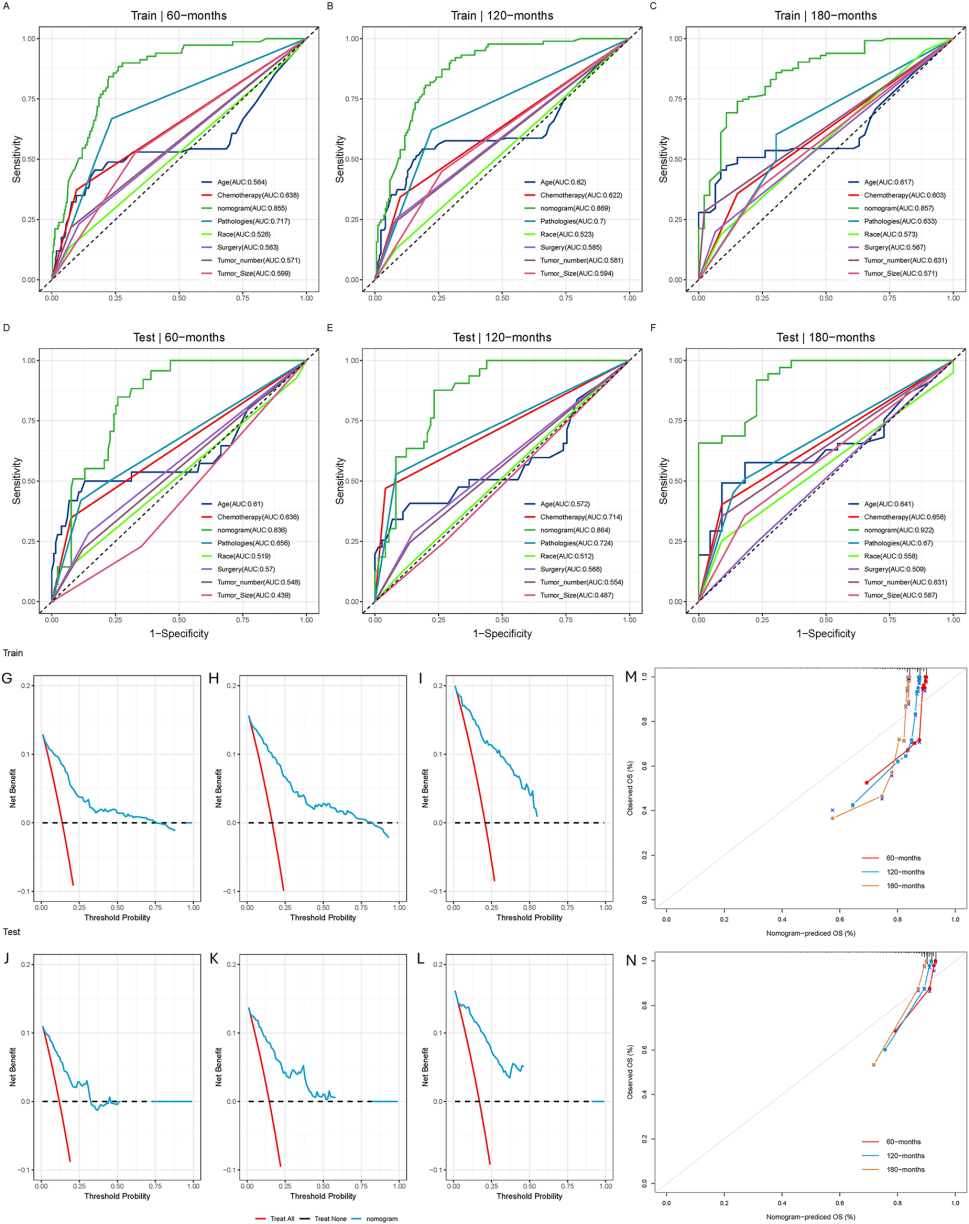
The receiver operating characteristic curves of nomogram and all independent predictors at 60- (**A**), 120- (**B**), and 180-months (**C**) in the training set and at 60- (**D**), 120- (**E**), and 180-months (**F**) in the testing set. The decision curve analysis of the nomogram at 60- (**G**), 120- (**H**), and 180-months (**I**) in the training set and the testing set (**J-L**). The calibration curve of the prognostic nomogram for patients with CPT in the training set (**M**) and the testing set (**N**).

These 808 patients with CPT were used to study prognostic factors. Of these, 566 patients were randomized into the training set and the remaining 242 patients were included in the test set. The chi-square test showed that there was no significant difference between the training and test sets (Table 3). A prognostic nomogram was established by combining clinical significance and statistical significance with the choice of age, tumor size, surgery, chemotherapy, tumor number, pathologies, and race (Fig. 2A). Based on the cut-off value of 0.168(Fig. 2B), we categorized all patients into low-risk and high-risk groups. The Kaplan–Meier survival curves showed that patients in the high-risk group had a worse prognosis than those in the low-risk group (Fig. 2C,D). In the training set, the AUCs at 60-, 120-, and 180-months were 0.855, 0.869 and 0.857, respectively (Fig. 3A–C). Afterward, we further compared the discrimination between the nomogram and the independent prognostic factors. The results indicated the AUC of nomogram was higher than AUCs of all independent factors in 60-, 120-, and 180-months. In the test set, the AUC at 60-, 120-, and 180-months were 0.836, 0.864 and 0.922, respectively (Fig. 3D–F). In the test set, we can find that the discriminative power of nomogram is also superior to all independent prognostic factors. The DCA curves show that nomogram has a good predictive effect on the survival probability of CPT patients (Fig. 3G–L). In addition, the calibration curve for survival probability also indicated a good consistency between nomogram predicted results and the actual outcome (Fig. 3M,N).

**Table 3:** Clinical and pathological features of patients diagnosed as CPT.

	Test	Train	*p* value
	(N = 242)	(N = 566)	
Time			
Mean (SD)	79.7 (60.5)	84.5 (62.5)	0.36
Median [Min, Max]	67.5 [1.00, 198]	74.0 [1.00, 249]	
Status			
Alive	212 (87.6%)	484 (85.5%)	0.43
Dead	30 (12.4%)	82 (14.5%)	
Age			
Mean (SD)	28.0 (25.4)	24.8 (23.8)	0.22
Median [Min, Max]	23.0 [0, 87.0]	17.0 [0, 86.0	
Radiation			
No	224 (92.6%)	529 (93.5%)	0.75
Yes	18 (7.4%)	37 (6.5%)	
Chemotherapy			
No	216 (89.3%)	492 (86.9%)	0.42
Yes	26 (10.7%)	74 (13.1%)	
Surgery			
No surgery	39 (16.1%)	79 (14.0%)	0.49
Surgery	203 (83.9%)	487 (86.0%)	
Sex			
Female	122 (50.4%)	286 (50.5%)	1
Male	120 (49.6%)	280 (49.5%)	
Race			
Black	15 (6.2%)	47 (8.3%)	0.54
Other	16 (6.6%)	41 (7.2%)	
White	211 (87.2%)	478 (84.5%)	
Pathologies			
ACPP	31 (12.8%)	57 (10.1%)	0.51
CPC	44 (18.2%)	103 (18.2%)	
CPP	167 (69.0%)	406 (71.7%)	
Tumor size			
< = 30 mm	158 (65.3%)	353 (62.4%)	0.48
> 30 mm	84 (34.7%)	213 (37.6%)	
Tumor number			
Multiple	20 (8.3%)	54 (9.5%)	0.66
Single	222 (91.7%)	512 (90.5%)	
Marital status			
Married	60 (24.8%)	134 (23.7%)	0.80
Other	182 (75.2%)	432 (76.3%)

## Discussion

CPTs comprise approximately 0.4–0.6% of all brain tumors^2,13^ and 1–4% of all brain tumors in children. Nausea, vomiting, irritability, headache, blurry vision, and seizures are the common clinical presentations. In infants and young children’s cases^8,11^, these clinical presentations are more serious because their skulls are not yet fully developed^14^. CPTs are rare, so previous studies have reported only limited cases of CPTs. We believe that this limitation may impede our understanding of the characteristics of patients with CPTs. To further explain the epidemiology and survival of CPTs, we performed a cohort study of 838 patients. Descriptive analysis was used to assess the distribution and tumor-related characteristics of the patients with CPTs. The nomogram was established to predict the prognosis of patients with CPT, which had satisfactory accuracy. This is one of the largest retrospective cohort studies of CPTs to date, and it may provide guidelines for neurosurgeons when treating this disease.

There have been previous studies^1,2,11^ of CPT using the SEER database. However, at that time, the database was not well established, with fewer CPT cases, and did not record patients’ chemotherapy situations. In recent years, the SEER database has been continuously updated with patient data, adding more cases of CPTs and chemotherapy-related information in 2023. Therefore, more data can be used to analyze and better understand this disease. We found a significant improvement in the OS of patients with CPTs compared to previous studies^1,2,15^, suggesting an overall improvement in the treatment of CPTs over time. In our study, histology types were found to be an important prognostic factor for OS, which is consistent with previously published findings^1,4,8,16,17^. We found that OS was superior for CPPs to ACPPs and for ACPPs to CPCs, although Merino et al.^18^ found no significant difference in the molecular characteristics of ACPPs and CPPs.

Through demographic studies, we found that patients with different pathology types had different age distributions. CPPs are more evenly distributed throughout all age groups, whereas CPCs occur in younger children, in agreement with previous studies’ findings^1,2,11,13^. For CPTs, black people had a worse prognosis than other people. Some reports^1,19^ noted that although race was the main factor affecting OS, it did not significantly affect the cause-specific survival. This may be related to socioeconomic factors. Studies looking at disparities in brain tumors suggest that black patients are less likely to be treated by high-volume healthcare providers than white patients, which may lead to poorer outcomes^19–21^. Differences in brain tumor survival between racial groups may also be related to differences in tumor biology, although the exact mechanisms remain incompletely understood. In addition, racial differences in drug metabolism and drug sensitivity may lead to further differences in prognosis. Addressing these issues is critical to achieving more equitable cancer outcomes. Patients with tumor sizes greater than 3 mm and multiple tumors have a worse prognosis, as well as more pronounced clinical symptoms. The effect of sex, marital status, and on prognosis was not statistically significant, Cannon and colleagues^1^ reached the same conclusion.

For patients with CPT of different pathologic types, surgery was chosen over radiotherapy or chemotherapy^1,2,9–11^. Bahar et al.^8^ concluded that all children with CPT should undergo GTR surgery. Although CPT is considered to be slow growing, there is a high risk of hydrocephalus or intracranial hemorrhage due to its specific primary site^2,8,11,22^. Without surgery, it is difficult to control the clinical symptoms of CPT. However, due to the vascularity and large size of CPT, today’s neurosurgeons still face great challenges in their quest to remove the tumor completely. In our univariate Cox regression analysis, surgery was prognostically favorable and statistically significant compared to no surgery, yet the prognosis of patients who underwent GTR versus STR did not show a significant difference. This may be attributed to the fact that the extent of resection of intracranial tumors is to some extent a subjective factor with no precisely defined criteria. Therefore, we combined GTR and STR for surgery and performed multivariate Cox regression analysis. The results showed that surgery had a significant effect on prognosis. Moreover, surgery still plays an irreplaceable role in alleviating the clinical symptoms of CPT^23^. Therefore, we believe that pursuing GTR surgery remains the first choice for the clinical treatment of CPT, which is consistent with the results of previous studies.

Except for surgery, the benefits of adjuvant chemotherapy and radiation therapy in patients remain controversial. We found that only a minority of patients with CPPs chose radiotherapy (2.9%) and chemotherapy (0.7%), whereas for CPCs, more patients chose chemotherapy (12.1%) over radiotherapy (6.6%). However, according to univariate Cox regression analysis, we found that radiotherapy and chemotherapy did not improve the survival rate of CPT patients. Even in the results of multivariate Cox regression analysis, the application of chemotherapy was unfavorable and statistically significant for prognosis, a result contrary to our clinical experience. This may be related to the selective bias of chemotherapy application. For example, in clinical practice, patients with more severe disease will be recommended by doctors to receive radiotherapy or chemotherapy; their acceptance rate of chemotherapy will also be higher because of their severe disease. Our guess can also be confirmed from the pathologic typing of the patients who received chemotherapy, the vast majority of which had the worse prognosis of CPC, accounting for 92.08% of all patients. While radiotherapy was not statistically significant in the results of multivariate Cox regression analysis. In addition, the reasons for the poor outcomes of chemotherapy on choroid plexus tumors, or even worse prognosis, we believe may be the following. Firstly, compared with other types of tumors, the biological characteristics of choroid plexus tumor cells may make them less sensitive to chemotherapy treatments, which may lead to poor therapeutic effects. Patients with choroid plexus tumors may experience serious toxic side effects after receiving chemotherapy, such as bone marrow suppression, liver, and kidney function impairment. These side effects may aggravate the patient’s condition and reduce the survival prognosis due to the generally younger age of the patients. If a patient receiving chemotherapy is diagnosed at a more advanced stage of the disease, that may result in a poorer prognosis. It is disappointing to conclude that chemotherapy does not benefit the prognosis of patients. We hope that clinical treatment planners will be aware of this and consider the patient’s condition to develop a personalized treatment plan rather than blindly applying chemotherapy. However, whether chemotherapy is truly ineffective against choroid plexus tumors also needs to be considered in the context of the type of chemotherapeutic agent used. Humanized animal models and further prospective clinical trials are needed to identify chemotherapeutic agents that are sensitive to choroid plexus tumors and have fewer toxic side effects. Existing studies have presented different views on whether radiotherapy should be taken for CPT patients with different pathologic subtypes. Farmer and colleagues^24^ suggested that children with ACPPs only undergo surgical resection, avoiding adjuvant radiotherapy. Although some studies did not find a significant survival advantage associated with radiation therapy, researchers have suggested performing radiotherapy after surgery for CPCs^2,6,25,26^. However, for patients with structural variation in TP53, Li et al.^25^ suggested not choosing radiation treatment. Mazloom et al.^15^ reported that patients with CPCs who received craniospinal irradiation (CSI) had better progression-free survival than those who received less CSI. A study^27^ noted that it is not recommended for patients younger than 3 years of age to choose radiation treatment because it has potential negative neurological sequelae in the developing brain. Overall, based on the available studies, there is not enough evidence to conclude that chemotherapy and radiotherapy are prognostically beneficial. Since most of the patients are children and adolescents, premature application of chemotherapy and radiotherapy may have a negative impact on their growth and development. Therefore, we recommend clinicians to carefully consider whether to apply radiotherapy or chemotherapy to patients^28^.

In our study, we established a prognostic nomogram for patients with CPT. The area of the box and the area under the curve represent the distribution of the data. This allows us to visualize the distribution of patients for each independent prognostic factor. By obtaining the data of several easily accessible variables on the nomogram of each CPT patient, the total score can be calculated. Patients with a total score greater than 0.186 have a high-risk rating for their condition. The probability of death within 180, 120, and 60 months can then be easily predicted by making a vertical line from the total score downward on the graph to intersect the three axes, thus providing guidance for further clinical treatment. This will make the individualized clinical decision and surveillance more accurate. CPT is a very rare disease in clinical practice, for this reason, clinicians can be overwhelmed when faced with decisions related to CPT prognosis^29^. Therefore, it is of great importance to explore the prognostic factors of patients with CPT. Although age, sex, type of pathology, extent of surgery, and application of radiotherapy have been reported as prognostic factors for CPT. To the present day, no CPT survival prediction model has been established, which means that the survival risk of CPT cannot be predicted by combining all predictive factors associated with CPT. The impact of distinct treatment strategies on the prognosis of pathologically staged CPP and CPC, respectively, was elucidated in the model developed by Bhutada et al.^30^. However, individual factors and tumor characteristics were absent. The results of our study indicate that individual factors (age, race), tumor characteristics (number of tumors, tumor size, pathology type), and treatment strategies (surgery and chemotherapy) are independent prognostic factors for CPT. To create a nomogram with a wider range of applicability, we included all these factors in the prediction model. Using the nomogram, clinicians can better explain to patients the benefits and risks of each prognostic factor and the corresponding prognostic outcomes. In addition, in patient-doctor communication, the nomogram can help patients understand their prognosis in a visual way, thereby improving their compliance with surgical treatment. Another improvement of the nomogram was that the discrimination of nomogram was confirmed higher than any single predictors, which also showed the importance of a comprehensive predictive model.

## Limitations

Retrospective studies often face limitations related to the inadequate handling of confounding variables. To control for confounders, we employed a commonly used method in clinical and epidemiological studies, selecting variables that were statistically significant (*p* < 0.05) in both univariate and multivariate Cox regression analyses (refer to Table 2 and Fig. 1J, respectively) as independent prognostic factors. We validated our model by plotting and calibrating ROC and DCA curves for both the training and test sets, demonstrating its reliability. Nevertheless, unveiling deeper associations between variables necessitates a larger, prospective, randomized, multicenter cohort study, a challenging endeavor due to the low incidence of choroid plexus tumors (CPT) which requires extensive collaboration across countries and healthcare organizations. In light of these challenges, we have developed a relatively robust prediction model using data from the SEER database to compensate for the current studies’ relative data scarcity.

However, our study has some limitations. First, the limited number of CPT patients may lead to a corresponding error. Second, the information collected in the SEER database was from the US region, which means that the conclusions drawn cannot fully represent the situation of CPT in other countries and regions. Third, this is a retrospective study, and selection bias is inevitable. Moreover, the number of patients with missing tumor sizes was large, making it difficult to perform quantitative analysis. At the same time, we do not know exactly which ventricle the tumor was located in. CPTs mainly affect the circulation of normal cerebrospinal fluid and cause a series of clinical symptoms. Therefore, the tumor size and location data are considered to have a critical impact on patient prognosis, which needs to be further studied by researchers in the future. We will follow up further prognostic studies of the disease and do external validation on this model with case data from other databases to ensure the reliability of the model.

## Conclusion

CPTs are rare intracranial tumors that frequently occur in children. Among all 3 histological types, CPCs had the worst prognosis. Age, race, pathologies, tumor size, tumor number, surgery, and the use of chemotherapy are independent prognostic factors in patients with CPT. Based on our analysis, radiotherapy and chemotherapy did not seem to provide more survival benefit to patients with CPT. Therefore, treatment should be individualized according to the patient’s condition. We developed a nomogram for predicting the prognosis of CPT patients, which can be an individual, convenient, and more intuitive visual tool. We suggest that more attention be paid to the treatment and prognosis of CPT patients. And we hope that more data from the SEER database will be available in the future for a more comprehensive analysis.

## Data Availability

Only publicly available data were used in our study, and data sources and handling of these data are described in the Materials and Methods. Further information is available from the corresponding author upon request.

## Abbreviations

ACPP: Atypical choroid plexus papilloma
AUC: Area under the curve
CPC: Choroid plexus carcinoma
CPP: Choroid plexus papilloma
CPT: Choroid plexus tumors
CSI: Craniospinal irradiation
DCA: Decision curve analysis
GTR: Gross total resection
OS: Overall survival
ROC: Receiver operating characteristic
SEER: Surveillance, epidemiology, and end results
STR: Subtotal resection
US: United States
WHO: World Health Organization

## References

[1] Cannon DM, Mohindra P, Gondi V, Kruser TJ, Kozak KR (2015). Choroid plexus tumor epidemiology and outcomes: Implications for surgical and radiotherapeutic management. J. Neurooncol..

[2] Gupta N (2003). Choroid plexus tumors in children. Neurosurg. Clin. N. Am..

[3] Li Y, Di C, Song S, Zhang Y, Lu Y, Liao J, Lei B, Zhong J, Guo K, Zhang N, Su S (2023). Choroid plexus mast cells drive tumor-associated hydrocephalus. Cell.

[4] Wolff JE, Sajedi M, Brant R, Coppes MJ, Egeler RM (2002). Choroid plexus tumours. Br. J. Cancer.

[5] Pienkowska M, Choufani S, Turinsky AL, Guha T, Merino DM, Novokmet A, Brudno M, Weksberg R, Shlien A, Hawkins C, Bouffet E, Tabori U (2019). DNA methylation signature is prognostic of choroid plexus tumor aggressiveness. Clin. Epigenetics.

[6] Lam S, Lin Y, Cherian J, Qadri U, Harris DA, Melkonian S, Jea A (2013). Choroid plexus tumors in children: A population-based study. Pediatr. Neurosurg..

[7] Faltermeier C, Chai T, Syed S, Lau N, Elkaim L, Ibrahim G, Wang A, Weil A, Bendel A, Fallah A, Tu A (2019). Survival of infants </=24 months of age with brain tumors: A population-based study using the SEER database. PLoS One.

[8] Bahar M, Hashem H, Tekautz T, Worley S, Tang A, de Blank P, Wolff J (2017). Choroid plexus tumors in adult and pediatric populations: the Cleveland Clinic and University Hospitals experience. J. Neurooncol..

[9] Mallick S, Benson R, Melgandi W, Rath GK (2017). Effect of surgery, adjuvant therapy, and other prognostic factors on choroid plexus carcinoma: A systematic review and individual patient data analysis. Int. J. Radiat. Oncol. Biol. Phys..

[10] Hosmann A, Hinker F, Dorfer C, Slavc I, Haberler C, Dieckmann K, Knosp E, Czech T (2019). Management of choroid plexus tumors—An institutional experience. Acta Neurochir. (Wien).

[11] Dudley RW, Torok MR, Gallegos D, Liu AK, Handler MH, Hankinson TC (2015). Pediatric choroid plexus tumors: Epidemiology, treatments, and outcome analysis on 202 children from the SEER database. J. Neurooncol..

[12] Thomas C, Soschinski P, Zwaig M, Oikonomopoulos S, Okonechnikov K, Pajtler KW, Sill M, Schweizer L, Koch A, Neumann J, Schuller U, Sahm F (2021). The genetic landscape of choroid plexus tumors in children and adults. Neuro Oncol..

[13] Gopal P, Parker JR, Debski R, Parker JC (2008). Choroid plexus carcinoma. Arch. Pathol. Lab. Med..

[14] Liu APY, Liu Q, Shing MMK, Ku DTL, Fu E, Luk CW, Ling SC, Cheng KKF, Kwong DLW, Ho WWS, Ng HK, Gajjar A (2020). Incidence and outcomes of CNS tumors in Chinese Children: Comparative analysis with the surveillance, epidemiology, and end results program. JCO Glob. Oncol..

[15] Mazloom A, Wolff JE, Paulino AC (2010). The impact of radiotherapy fields in the treatment of patients with choroid plexus carcinoma. Int. J. Radiat. Oncol. Biol. Phys..

[16] Wolff JE, Van Gool SW, Kutluk T, Diez B, Kebudi R, Timmermann B, Garami M, Sterba J, Fuller GN, Bison B, Kordes UR (2022). Final results of the choroid plexus tumor study CPT-SIOP-2000. J. Neurooncol..

[17] Thomas C, Metrock K, Kordes U, Hasselblatt M, Dhall G (2020). Epigenetics impacts upon prognosis and clinical management of choroid plexus tumors. J. Neurooncol..

[18] Merino DM, Shlien A, Villani A, Pienkowska M, Mack S, Ramaswamy V, Shih D, Tatevossian R, Novokmet A, Choufani S, Dvir R, Ben-Arush M (2015). Molecular characterization of choroid plexus tumors reveals novel clinically relevant subgroups. Clin. Cancer Res..

[19] Moore KJ, Moertel CL, Williams LA (2022). Minority children experience a higher risk of death from many central nervous system tumor types even after accounting for treatment received: A national cancer database analysis. Cancer.

[20] Haizel-Cobbina J, Spector LG, Moertel C, Parsons HM (2021). Racial and ethnic disparities in survival of children with brain and central nervous tumors in the United States. Pediatr. Blood Cancer.

[21] Wellbrock M, Voigt M, Ronckers C, Grabow D, Spix C, Erdmann F (2024). Registration, incidence patterns, and survival trends of central nervous system tumors among children in Germany 1980–2019: An analysis of 40 years based on data from the German Childhood Cancer Registry. Pediatr. Blood Cancer.

[22] Lin H, Leng X, Qin CH, Du YX, Wang WS, Qiu SJ (2019). Choroid plexus tumours on MRI: Similarities and distinctions in different grades. Cancer Imaging.

[23] Faramand A, Kano H, Niranjan A, Atik AF, Lee CC, Yang HC, Mohammed N, Liscak R, Hanuska J, Tripathi M, Kondziolka D, Sheehan J (2021). Stereotactic radiosurgery for choroid plexus tumors: A report of the international radiosurgery research foundation. Neurosurgery.

[24] Browne-Farmer C, Hazrati LN, Mamatjan Y, Zadeh G, Dirks P, Rutka J, Malkin D, Bouffet E, Huang A, Tabori U, Ramaswamy V, Bartels U (2021). Paediatric atypical choroid plexus papilloma: Is adjuvant therapy necessary?. J. Neurooncol..

[25] Li Y, Liu H, Li T, Feng J, He Y, Chen L, Li C, Qiu X (2021). Choroid plexus carcinomas with TP53 germline mutations: Management and outcome. Front. Oncol..

[26] Bishop AJ, McDonald MW, Chang AL, Esiashvili N (2012). Infant brain tumors: Incidence, survival, and the role of radiation based on surveillance, epidemiology, and end results (SEER) Data. Int J Radiat. Oncol. Biol. Phys..

[27] Sun MZ, Oh MC, Ivan ME, Kaur G, Safaee M, Kim JM, Phillips JJ, Auguste KI, Parsa AT (2014). Current management of choroid plexus carcinomas. Neurosurg. Rev..

[28] Ruiz-Garcia H, Huayllani MT, Incontri D, Whaley JJ, Marenco-Hillembrand L, Ebot J, Chaichana KL, Sheehan J, Quinones-Hinojosa A, Trifiletti DM (2020). Intraventricular choroid plexus tumors: Clinical characteristics and impact of current management on survival. J. Neurooncol..

[29] Zuo P, Mai Y, Jiang Z, Zhang B, Wang Y, Zhang M, Wu Z, Zhang J, Zhang L (2023). Primary adult choroid plexus carcinomas: A single-center experience with a systematic review. Front. Oncol..

[30] Bhutada AS, Adhikari S, Cuoco JA, In A, Rogers CM, Jane JA, Marvin EA (2024). Prognostic factors and nomogram for choroid plexus tumors: A population-based retrospective surveillance, epidemiology, and end results database analysis. Cancers (Basel).

